# UNCLES: method for the identification of genes differentially consistently co-expressed in a specific subset of datasets

**DOI:** 10.1186/s12859-015-0614-0

**Published:** 2015-06-04

**Authors:** Basel Abu-Jamous, Rui Fa, David J. Roberts, Asoke K. Nandi

**Affiliations:** 1 Department of Electronic and Computer Engineering, Brunel University London, Uxbridge, Middlesex UB8 3PH UK; 2 National Health Service Blood and Transplant, Oxford, OX3 9BQ UK; 3 Radcliffe Department of Medicine, University of Oxford, John Radcliffe Hospital, Oxford, OX3 9DU UK; 4 Department of Mathematical Information Technology, University of Jyväskylä, Jyväskylä, Finland

**Keywords:** Genome-wide analysis, Consistent co-expression, Bi-CoPaM, UNCLES, Multiple datasets analysis

## Abstract

**Background:**

Collective analysis of the increasingly emerging gene expression datasets are required. The recently proposed *binarisation of consensus partition matrices (Bi-CoPaM)* method can combine clustering results from multiple datasets to identify the subsets of genes which are consistently co-expressed in all of the provided datasets in a tuneable manner. However, results validation and parameter setting are issues that complicate the design of such methods. Moreover, although it is a common practice to test methods by application to synthetic datasets, the mathematical models used to synthesise such datasets are usually based on approximations which may not always be sufficiently representative of real datasets.

**Results:**

Here, we propose an unsupervised method for the *unification of clustering results from multiple datasets using external specifications (UNCLES)*. This method has the ability to identify the subsets of genes consistently co-expressed in a subset of datasets while being poorly co-expressed in another subset of datasets, and to identify the subsets of genes consistently co-expressed in all given datasets. We also propose the *M-N scatter plots* validation technique and adopt it to set the parameters of UNCLES, such as the number of clusters, automatically. Additionally, we propose an approach for the synthesis of gene expression datasets using real data profiles in a way which combines the ground-truth-knowledge of synthetic data and the realistic expression values of real data, and therefore overcomes the problem of faithfulness of synthetic expression data modelling. By application to those datasets, we validate UNCLES while comparing it with other conventional clustering methods, and of particular relevance, biclustering methods. We further validate UNCLES by application to a set of 14 real genome-wide yeast datasets as it produces focused clusters that conform well to known biological facts. Furthermore, *in-silico*-based hypotheses regarding the function of a few previously unknown genes in those focused clusters are drawn.

**Conclusions:**

The UNCLES method, the M-N scatter plots technique, and the expression data synthesis approach will have wide application for the comprehensive analysis of genomic and other sources of multiple complex biological datasets. Moreover, the derived *in-silico*-based biological hypotheses represent subjects for future functional studies.

**Electronic supplementary material:**

The online version of this article (doi:10.1186/s12859-015-0614-0) contains supplementary material, which is available to authorized users.

## Background

Some genes’ expression profiles might be found well correlated in a single microarray dataset for many reasons other than that they are co-regulated or that they function within the same pathway [1–3]. On the other hand, consistent co-expression of the same subset of genes across many independent datasets may indeed indicate a higher likelihood of co-regulation and/or linked function [1, 2, 4–6]. Some studies have used a core subset of genes that are well known to participate in the target pathway as a template, and then many microarray datasets were mined for the genes that are consistently co-expressed with that template of genes [2, 6]. One drawback of this approach is that it cannot be applied without the availability of a starting template of co-expressed genes. Another significant shortcoming is that this approach is not able to discover other subsets of genes that are also consistently co-expressed in the same datasets but with different profiles from the starting template.

Unsupervised clustering methods do not require a starting template. Conventional unsupervised clustering algorithms, such as k-means [7], hierarchical clustering [8], self-organising maps [9], and many others, tackle the problem of identifying the genes that are co-expressed within any single dataset. In contrast we have recently proposed the binarisation of consensus partition matrices (Bi-CoPaM) method [10], which has the unique ability to address, in an unsupervised way, the research question: which are the subsets of genes that are consistently co-expressed over a set of genome-wide (or filtered) datasets? Those datasets could have been generated under different conditions and biological contexts, and even from different species [11].

Other types of external specifications can be proposed to scrutinise the clustering results from multiple datasets. For instance, it is very useful to identify the subsets of genes specifically consistently co-expressed in one specific subset of datasets while being poorly consistently co-expressed in another subset of datasets. Although, to the authors’ knowledge, this research question has not been answered in an unsupervised way previously, it has been raised and discussed implicitly and explicitly in many studies [2–4, 12–14]. However, biclustering methods, such as Cheng and Church (CC) [15], Plaid [16], Bimax [17], and others, mine a data matrix for the rows (corresponding to genes) that show consistent co-expression across all or some of the matrix columns (corresponding to samples). Although such methods were designed to mine a single dataset, multiple datasets may be concatenated to provide a single data matrix that is fed to biclustering analysis.

Despite the Bi-CoPaM’s successful application in some studies, it has been used where the number of clusters is known based on *a priori* knowledge [18, 19]. Automatic setting for the number clusters as well as the other parameters of the Bi-CoPaM was evident while proposing the Bi-CoPaM but has not been resolved yet [10]. Another unresolved issue is the design of a validation technique for the tunable results of the Bi-CoPaM [10]. The problem of requiring a manually pre-set number of clusters is common to most clustering methods and has been discussed thoroughly in the literature but with no solution that suits the nature of the Bi-CoPaM [10, 20–22].

In this paper, we propose a new method named as *the unification of clustering results from multiple datasets using external specifications (UNCLES)*. This method unifies the clustering results from multiple datasets under one of two types of external specifications. The first unifies the clustering results from multiple datasets to identify the subsets of genes consistently co-expressed over all of the given datasets. The second type aims at unifying such clustering results in order to identify the subsets of genes consistently co-expressed over one subset of datasets while being poorly co-expressed over another subset of datasets. We also present a novel validation technique, based on the proposed M-N scatter plots, which addresses the problem of setting the proper number of clusters (K) as well as the tuning parameters for both methods, the recently proposed Bi-CoPaM and the novel UNCLES.

## Methods

### Synthetic data generation

We have selected the datasets under the GEO accession numbers GSE18057 [23], GSE10124 [24], GSE12736 [25], and GSE9386 [26] whose clustering analysis have been previously provided by the relevant references. The four datasets were derived from the species Oryza sativa (Asian rice), Xenopus laevis (African clawed frog), Homo sapiens (human), and Zea mays (maize), respectively, and their respective numbers of samples are 36, 6, 16, and 24. We have produced six synthetic datasets, labelled as P1, P2, P3, N1, N2, and N3 based on these four real datasets where P1 and P2 are respectively based on the first 18 and the last 18 samples of GSE18057, P3 is based on GSE10124, N1 is based on GSE12736, and N2 and N3 are based on the first and the last twelve samples of GSE9386 respectively.

**Fig. 1:**
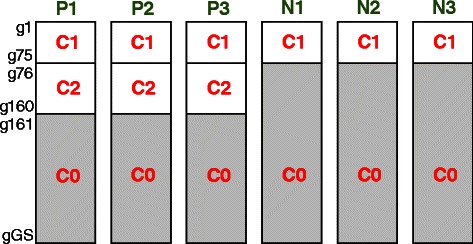
The structure of the six synthetic microarray datasets. The cluster C1 (*g1 to g75*) includes genes consistently co-expressed over all of the six datasets, and the cluster C2 (*g76 to g160*) includes genes consistently co-expressed only in the positive set of datasets (*P1, P2, and P3*) while being poorly co-expressed in the negative set of datasets (*N1, N2, and N3*). The rest of the genome (C0) includes genes poorly co-expressed everywhere

**Fig. 2:**
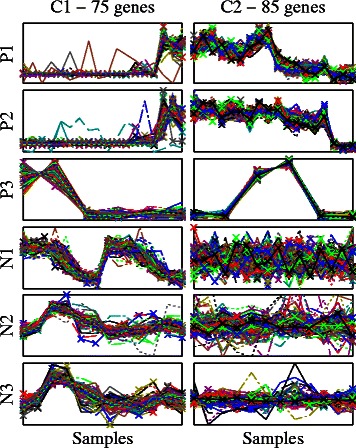
Synthetic data ground truth clusters *C1* and *C2* expression profiles. Each plot in this grid of plots shows the normalised expression profiles of the 75 and 85 genes respectively included in the ground truth clusters *C1* and *C2* in each of the six synthetic datasets. The horizontal axis is the samples axis whose range in each subplot is equal to the number of samples of the corresponding dataset. The vertical axis is the normalised expression value. Note that *C1* is consistently co-expressed in all of the six datasets while *C2* is only consistently co-expressed in the positive datasets *P1, P2,* and *P3*

The gene names/probe identifiers of the original datasets were omitted and the artificial gene names g1 to g*GS* were used instead, where *GS* is the artificial genome size. Therefore the i^th^ gene (gi) in each of the six synthetic datasets is considered as the same gene whose expression profile is assumed to be measured in six different microarray datasets. In each of the six datasets, the artificial genes g1 to g75 were selected from one of the defined clusters in the relevant study (cited in the previous paragraph), i.e. the profiles of those 75 genes in each of the datasets were previously confirmed to be co-expressed in the literature; these genes have been labelled as the cluster C1 (Figs. 1 and 2). The 85 genes g76 to g160 were selected in the same way but only in the positive datasets P1, P2, and P3, and have been labelled as the cluster C2 (Figs. 1 and 2). The rest of the genome, i.e. g161 to g*GS* in P1, P2, and P3, and g76 to g*GS* in N1, N2, and N3, were randomly selected from the genes excluded from clustering analysis in the relevant studies for being not differentially expressed, i.e. poorly co-expressed everywhere, and have been labelled as C0 (Fig. 1). We have generated data with the genome sizes (*GS*) of 1200, 2000, 3000, 5000, and 7000 genes respectively to create five sets of datasets.

For more realistic modelling, some genes (less than ten to a few tens) from C0 in each of the datasets are co-expressed with either C1 or C2 in the specific dataset in which they occur without being consistently co-expressed over the rest of the datasets, i.e. the genes of C1 are consistently co-expressed in all of the six datasets, but in each of the datasets individually, there are few more genes that are also co-expressed with those 75 but that differ from one dataset to another. The same applies to C2 in the positive datasets.

All of the 30 produced datasets (six datasets for each of the five genome sizes) are provided in Additional files 1, 2, 3, 4, and 5 alongside the membership of genes in C1 or C2.

### Bi-CoPaM

Binarisation of consensus partition matrices (Bi-CoPaM) which has been recently proposed by Abu-Jamous et al. [10], is applied to a set of transcriptomic datasets (e.g. microarray datasets). This method does not combine the datasets themselves; rather it performs clustering over each one of the datasets independently in the first stage. Therefore, within a dataset all genes are homogeneous in that they have the same experimental design, e.g. number of samples/time points and distances between time points. In a later stage of the processing, the resulting partitions from each of the datasets are combined based on memberships and independent of the time profiles of the genes in their datasets, to produce one set of clusters. This approach of projecting the datasets into this invariant space of membership by clustering allows us to analyse multiple heterogeneous datasets collectively. Moreover, the datasets do not have to be time-series, that is, the horizontal axis of the gene expression profile may not represent time. For example, the data points (samples) may represent samples from different types of tissues instead. In any case, each dataset is clustered independently, and when the resulting partition matrices are combined afterwards, they are invariant to the aforementioned variables and factors.

Bi-CoPaM is applied by following the following four main steps:

1. Individual partition generation: a partition (clustering result) is generated for the same set of genes by using one clustering algorithm on a selected dataset. By applying C different clustering methods to L different datasets measuring the expression for the same set of genes, *R* = *C* × *L* partitions are generated. The same number of clusters (K) should be used for all of these partitions.
2. Relabelling: each cluster from any individual partition is mapped to its most similar cluster from each of the other individual partitions. The clusters in each partition are accordingly permutated such that the clusters mapped to each other are aligned.
3. Fuzzy consensus partition matrix (CoPaM) generation: the fuzzy CoPaM is the average partition of the relabelled partitions. A gene’s fuzzy membership value in a cluster in the CoPaM matrix represents the ratio of times in which this gene has appeared in that particular clusters to the total number of individual partitions.
4. Binarisation: the fuzzy CoPaM is binarised to obtain a binary consensus partition matrix by using one of six proposed binarisation techniques.

We have used one of the six binarisation techniques originally proposed by Abu-Jamous and colleagues [10], which is the difference threshold binarisation (DTB). Based on the fuzzy values in the CoPaM matrix, DTB assigns a gene to the cluster in which it has its maximum fuzzy membership only if the difference between it and its membership in the closest competitor cluster is not less than the parameter δ. The gene is left unassigned from all of the clusters otherwise. The value of δ can range from zero to unity. When δ is zero, each gene is assigned to the cluster in which it has its maximum membership, therefore no genes are unassigned from all of the clusters, and the resulting clusters are complementary clusters that include the entire genome. When δ is equal to one, the gene is assigned to a cluster only if its fuzzy membership value in that cluster is equal to one, which only happens when all of the individual partitions have included that gene in that particular cluster consensually. Thus, δ is a tuning parameter which tunes the tightness of the clusters from being complementary clusters at (δ = 0) to the tightest case which leaves most of the genes in the genome unassigned from all of the clusters at (δ = 1).

It is worth noting that this method, as described, does not combine the datasets themselves; it rather combines the partitions resulting from clustering each dataset separately by various clustering methods. Therefore, the datasets maybe heterogeneous in terms of the number of samples (e.g. time-points), distances between time points in time-series datasets, number of channels in the microarray chip, laboratory, year, conditions, biological context, technology (microarrays versus next-generation sequencing (NGS)), and other factors. The key aspect that has to be common between those datasets is that they measure the expression (or any other quantity) for the same set of genes.

### Uncles

*The unification of clustering results from multiple datasets using external specifications (UNCLES)* is a novel method which we propose in this paper. Although the types of external specifications which would be proposed can be many, we propose two types of external specifications in this study:

*Type A:* the multiple datasets are mined for the subsets of genes consistently co-expressed in all of them. The Bi-CoPaM method [10] can be configured to achieve this objective by considering the *difference threshold binarisation (DTB)* technique with the tuning parameter δ ∊ [0, 1].

*Type B:* the multiple datasets are split into two subsets of datasets, the positive subset (S^+^) and the negative subset (S^−^). These are then mined for the subsets of genes consistently co-expressed in S^+^ while being poorly consistently co-expressed in S^−^. This is novel to the study.

**Fig. 3:**
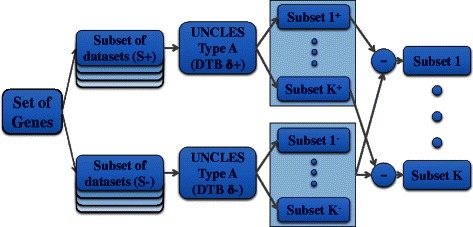
Flow chart summary for UNCLES with type B of external specifications

A flow chart for type B is shown in Fig. 3. First, UNCLES type A is applied to each of the two subsets of datasets, S^+^ and S^−^, separately by considering DTB binarisation with the parameters δ^+^ and δ^−^, respectively. Then, all of the genes which have been assigned to some cluster in the results of analysing the negative subset of datasets (S^−^) are unassigned from all of the clusters in the results of analysing the positive subset of datasets (S^+^). The resulting clusters are said to be generated at the parameter pair of (δ^+^, δ^−^).

The parameter δ^+^ controls how tight the clusters should be in the S^+^ datasets for their genes to be included in the result while the parameter δ^−^ controls how tight the clusters should be in the S^−^ datasets for their genes to be excluded. Therefore, the widest clusters are generated at the pair (0, 1) and the tightest clusters are generated at (1, ε) where ε is a very small real positive number. At the pair (1, 0), or any pair (δ^+^, 0), the clusters are totally empty, because, when δ^−^ is equal to zero, all of the genes are excluded from the clusters. Hence we can consider (1, ε) as the tightest non-trivial case.

### M-N scatter plot

In this paper, we propose the *MSE-related metric (M) - number of genes (N)*, i.e. the *(M-N)*, scatter plots technique to select the best cluster(s) out of the pool of clusters generated by UNCLES at different δ or (δ^+^, δ^−^) values as well as when different numbers of clusters (K) are used. This technique aims at minimising the dissimilarity between genes’ profiles in a cluster while maximising the number of genes included in it.

Given any dataset, the mean-squared error (MSE) metric for the *k* ^*th*^ cluster (*C*_*k*_) is:

1 $$ MS{E}_{cluster(k)}=\frac{1}{D\cdot {N}_k}{\displaystyle \sum_{x_i\in {C}_k}{\left\Vert {x}_i-{z}_k\right\Vert}^2}, $$

where *D* is the number of dimensions of the datasets, i.e. time- or data-points, *N*_*k*_ is the number of genes in the *k* ^*th*^ cluster, {*x*_*i*_} is the set of normalised expression profiles of genes included in the *k* ^*th*^ cluster, and *z*_*k*_ is the average expression profile of the genes included in the *k* ^*th*^ cluster.

The MSE-related metric (*M*) is defined as:

1. Type A: the average of the MSE values based on all of the datasets.
2. Type B: the average MSE values based on the S^−^ datasets subtracted from the average MSE values based on the S^+^ datasets.

For both types A and B, the MSE-related metric should be minimised to obtain better clusters.

**Fig. 4:**
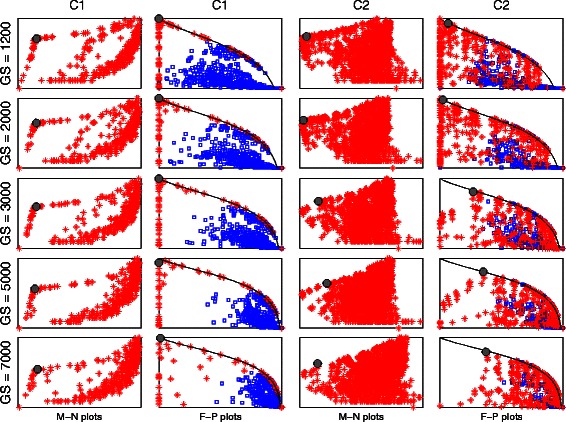
*M-N* and *F-P* scatter plots of the synthetic data clusters *C1* and *C2* generated by UNCLES and by other methods. The selected clusters in the M-N plots are marked by solid grey circles, and their corresponding points in the *F-P* plots are marked by solid grey circles as well. The red stars in any of the *M-N* or *F-P* plots represent the clusters produced by the UNCLES method while the blue squares in the *F-P* plots represent the clusters produced by the other methods

The M-N scatter plot is a plot on which the clusters are scattered, whose vertical axis is the logarithm of the number of genes included in a cluster (*N*), and whose horizontal axis is the MSE-related metric. Examples of M-N scatter plots are in Fig. 4 (first and third columns). The best cluster, based on our proposed technique, is that whose point on the M-N scatter plot is closest in distance to the top left corner after scaling both axes to have the same length.

Both types of UNCLES need the number of clusters (K) to be pre-set and fixed. The M-N scatter plot technique solves this problem by scattering all of the clusters generated by UNCLES by using many K values at all of the considered δ and (δ^+^, δ^−^) values on the same plot, and then selecting the best single cluster of all of those clusters.

If more than one best cluster is needed to be selected, after selecting the first best cluster as described, all of the scattered clusters on the plot which share some genes with that selected cluster are removed, and the closest cluster to the top left corner amongst the remaining clusters is selected as the second best cluster. This process of selection is repeated until the researcher obtains the desired clusters. One possible termination criteria can be that the next step’s cluster is significantly farther than the previous one from the top left corner of the M-N plot, and therefore there is a gap in quality. This is thoroughly demonstrated in the analysis of Figs. 9 and 10.

### F-P scatter plot

We propose a technique to evaluate the resulting clusters while knowing the ground truth by using the *false-positive-rate (F) - scaled-p-value (P)*, i.e. the (*F-P*) scatter plot. This technique has been used in this study to validate the results of our proposed UNCLES method and to validate our proposed M-N scatter plots technique. For the F-P scatter plot to be applicable, the ground truth must be known, and this is the case in our analysis of the set of synthetic datasets in this study.

If the objective is to discover a subset of *m* genes from a genome which contains *M* genes, and the adopted method discovered *N* genes, *n* of which are true-positives, i.e. members of the objective subset, then the p-value is defined as the probability of obtaining such a result or a better one randomly. This is expressed as:

2 $$ \mathrm{p}\hbox{-} \mathrm{value}={\displaystyle \sum_{\mathrm{j}=\mathrm{n}}^{\mathrm{N}}\left(\frac{\mathrm{N}!}{\mathrm{j}!\left(\mathrm{N}\hbox{-} \mathrm{j}\right)!}\right)\times {\left(\frac{\mathrm{m}}{\mathrm{M}}\right)}^{\mathrm{j}}\times {\left(\frac{\mathrm{M}\hbox{-} \mathrm{m}}{\mathrm{M}}\right)}^{\mathrm{N}\hbox{-} \mathrm{j}}}. $$

We define the scaled p-value as the ratio of the logarithm of the p-value to the logarithm of the best theoretically possible p-value at the given genome size. This is expressed as:

3 $$ \mathrm{scaled}\ \mathrm{p}\hbox{-} \mathrm{value}=\frac{ \log \left(\mathrm{p}\hbox{-} \mathrm{value}\right)}{ \log {\left(\raisebox{1ex}{$\mathrm{m}$}\!\left/ \!\raisebox{-1ex}{$\mathrm{G}\mathrm{S}$}\right.\right)}^{\mathrm{m}}}. $$

The scaled p-value ranges from unity for the theoretically ideal result to zero for clusters which do not include any of the true-positive genes.

Scaled p-values cannot capture the rate of false-positive discoveries in the cluster under evaluation and they might give better scores for clusters with very high false-positive rates if they were significantly larger than other clusters with much better false-positive rates. To capture this fact, we propose using F-P scatter plots to visualise the clusters while scattered on a plane consisting of both dimensions, false-positive rates (*F*) and scaled p-values (*P*). Both dimensions range between zero and unity.

Examples of F-P scatter plots are in Fig. 4 (second and fourth columns). The best theoretically possible cluster occurs on the top left corner of the plot of a scaled p-value of one and zero false-positives. The continuous black curve marks the zero false-negatives cases, and represents the theoretical upper limit for scaled p-values at any fixed FPR value.

### Statistical comparison between clustering methods based on F-P plots

While comparing two methods, clusters that have at least one true positive member are identified. Then, the closest 50 % of these clusters to the top-left corner of the corresponding F-P plot are considered for a *t*-test. After that, *t*-test is applied to test if the two subsets of distances are significantly different from each other. The generated statistics are the mean (μ) of the signed differences between distances, its standard deviation (σ), and the p-value. The mean of the signed differences ranges from $-\sqrt{2}$ to $\sqrt{2}$ because the diameter of the F-P plot is $\sqrt{2}$. Closer values to $-\sqrt{2}$ indicate that the clusters generated by the first method have smaller distances from the top left corner of the F-P plot and therefore are better, while the opposite is true when the values are closer to $\sqrt{2}$. Mean values closer to zero indicate that both methods’ results are similar to each other.

## Results

We have performed two sets of experiments in order to demonstrate the usefulness of the UNCLES method. The first set uses a set of six synthetic datasets generated by merging controlled parts of real datasets to preserve real datasets statistics (i.e. contain real measured values), and the second set of analyses uses 14 real budding yeast datasets.

### Synthetic data analysis

Synthetic datasets are commonly used in the validation of new computational methods as their ground truth is known and controlled beforehand. Many methods exist in the literature to model microarray expression data while considering different variables such as noise and degraded synchronisation, and are then used to generate synthetic datasets [27–30]. We have followed a different procedure to overcome these concerns, and to produce synthetic datasets that preserve the statistics of real datasets. As detailed in Methods, we have produced five sets of datasets with the genome sizes (*GS*) of 1200, 2000, 3000, 5000, and 7000 genes respectively. Each of the five sets includes six datasets labelled as P1, P2, P3, N1, N2, and N3. The synthetic datasets include one cluster with 75 genes, C1, that is consistently co-expressed in all of the six datasets, another cluster with 85 genes, C2, that is specifically consistently co-expressed in the positive datasets while being poorly co-expressed in the negative datasets, and the rest of the genome, C0, that is poorly consistently co-expressed everywhere (Fig. 2). All of the produced synthetic datasets are available in Additional files 1, 2, 3, 4, and 5.

#### Experimental setup

We have applied UNCLES to each of the five sets of synthetic datasets generated with the five different genome sizes (*GS*). Each of those sets of datasets has been considered with all of the numbers of clusters (K) of 4, 8, 12, 16, 20, and 25. We have applied UNCLES with both external specifications types A and B (see Methods). Type A aims at identifying the subsets of genes consistently co-expressed over all of the datasets, and type B aims at identifying the subsets of genes specifically consistently co-expressed in the positive set of datasets P1, P2, and P3, while being poorly consistently co-expressed in the negative set of datasets N1, N2, and N3. The used DTB δ values [10] for UNCLES type A are zero to unity with steps of 0.1, and the (δ^+^, δ^−^) pair values for the novel UNCLES type B are all possible pairs while ranging each of the δ values from zero to unity with steps of 0.1.

The individual clustering methods which have been used to produce the initial partitions fed to the following steps of UNCLES are k-means with KA initialisation [7], self-organising maps (SOMs) [9], and hierarchical clustering (HC) with Ward’s linkage [8]. Note that k-means mines for spherical clusters, SOMs consider competition between clusters while distributing their models (nodes) over a grid with defined spatial relations, and HC considers a hierarchical structure with a set of nested clusters that are joined or split based on the level of required resolution. In other words, we have considered three very popular methods belonging to three different families of clustering methods to maximise the diversity in clustering criteria and therefore increase the significance of the methods’ agreement, i.e. consensus.

Prior to clustering, it is important to ensure that the datasets are normalised appropriately. As per the studies from which the datasets were taken, the datasets P1, P2, P3, and N1 are based on one-channel microarray platforms and were normalised by quantile normalisation [23–25], while the datasets N2 and N3 are based on two-channel microarray platforms and were normalised by background subtraction, print-tip loess normalisation (within-array normalisation), and then between-array scaling normalisation [26]. Adopting these normalisation methods complies with the recommendation by the literature, such as the review by Roberts [31].

We also compared our results with the results of applying other methods to the same datasets. We have tested k-means with KA initialisation [7], self-organising maps (SOMs) [9], hierarchical clustering (HC) with Ward’s linkage [8], and the ensemble clustering method relabeling and voting [32]. These methods were applied separately to each of the six datasets within each of the five sets of datasets at the adopted genome sizes (*GS*) 1200 to 7000 and by considering the ten K values 4, 8, 12, 16, 20, 25, 50, 75, 100, and 125. The reason for using high K values for those methods, as opposed to UNCLES, is that those methods do not possess the unique feature of our method, which is the ability to tune the results to obtain tighter clusters while leaving most of the genes unassigned to any cluster. In other words, for those methods to obtain clusters of sizes that are comparable to the sizes of the ground truth clusters (75 and 85), high K values are needed.

#### UNCLES results

The perfect result of 100 % specificity and 100 % sensitivity would be obtained if the cluster C1 is discovered by type A of UNCLES, and that the cluster C2 is discovered by type B. For any single set of datasets, i.e. for a specific genome size (*GS*), there are 935 individual clusters generated by type A by considering all of the K and the δ values, and there are 10,285 individual clusters generated by type B by considering all of the K and the (δ^+^, δ^−^) pair values. Each of the other four clustering methods has generated 2,610 individual clusters by considering all of the K values; remembering that those methods have been applied to the six datasets separately, not collectively.

M-N scatter plots (see Methods) for each of the considered genome sizes for UNCLES types A and B are shown in Fig. 4 (the first and the third columns) while marking the selected best cluster in each case with a solid grey circle. To validate our approach, we have also shown the ground-truth-dependent F-P scatter plots (see Methods) for each of these cases in the second and the fourth columns (Fig. 4). The selected clusters based on the M-N scatter plots are also marked on the F-P plots with solid grey circles.

The first, most relevant and most interesting observation is that in both types of external specifications A and B, i.e. for clusters C1 and C2, and for all of the genome sizes considered (*GS*), the clusters selected based on the ground-truth-independent approach scored the best (M-N plots), or very close to the best, scores in the ground-truth-dependent approach (F-P plots) (Fig. 4). This not only proves the ability of UNCLES to find the clusters of genes that meet each of the proposed types of external specifications A and B, but also proves the validity of using the M-N scatter plots approach to select the best clusters from the methods’ results.

All of the clusters generated by the other four clustering methods with which we compare our method, and based on all of the datasets and K values, are scattered on the sub-plots of the second and the fourth columns in Fig. 4. For both C1 and C2, all of the clusters generated by the other four methods, even at their best, lag significantly behind many of the clusters generated by our method including the ones selected by the M-N plot approach (F-P plots in Fig. 4 and the third column in Table 1). On the other hand, there is no similarly significant difference among these four methods (the fourth column in Table 1).

**Table 1:** Clustering methods’ performance comparison

C	*GS*	UNCLES versus closest competitor^*^	Most separated pair of other methods^*^
C1	1,200	−0.81 ± 0.15 (9.3 × 10^−61^) [HC]	−0.13 ± 0.17 (1.5 × 10^−10^) [HC, RV]
	2,000	−0.88 ± 0.17 (7.3 × 10^−55^) [HC]	−0.15 ± 0.18 (1.7 × 10^−11^) [HC, RV]
	3,000	−0.93 ± 0.15 (1.6 × 10^−68^) [HC]	−0.12 ± 0.16 (2.5 × 10^−11^) [SOMs, RV]
	5,000	−0.92 ± 0.15 (7.6 × 10^−66^) [HC]	−0.09 ± 0.14 (1.9 × 10^−8^) [SOMs, RV]
	7,000	−0.77 ± 0.15 (3.6 × 10^−54^) [SOMs]	−0.08 ± 0.12 (2.9 × 10^−9^) [SOMs, RV]
C2	1,200	−0.93 ± 0.15 (<10^−255^) [SOMs]	−0.04 ± 0.14 (5.8 × 10^−7^) [SOMs, RV]
	2,000	−0.92 ± 0.17 (<10^−255^) [HC]	−0.04 ± 0.12 (5.0 × 10^−7^) [HC, RV]
	3,000	−0.60 ± 0.15 (6.3 × 10^−244^) [HC]	−0.03 ± 0.11 (6.7 × 10^−5^) [HC, RV]
	5,000	−0.55 ± 0.13 (1.1 × 10^−234^) [HC]	−0.02 ± 0.09 (2.0 × 10^−4^) [HC, RV]
	7,000	−0.48 ± 0.13 (4.8 × 10^−219^) [HC]	−0.02 ± 0.09 (1.3 × 10^−3^) [HC, RV]

*The format of the entries in these two columns is: μ ± σ (*p-value*) [method(s)]. The closest competitor to UNCLES is the one with the largest p-value while the most significantly separated pair of other clustering methods is the pair with the smallest p-value. See Methods for details

The black continuous curves in the F-P plots in Fig. 4 mark the upper theoretical boundary of the scaled p-value at any given FPR value; this happens when the clustering method does not miss any of the target genes, i.e. at zero false-negatives. The top left corner represents the ideal case which is when the discovered cluster has exactly all of the target genes (75 genes for C1 and 85 genes for C2). For any fixed cluster’s size, the best case is to have no false-positives if the cluster’s size is less than or equal to the number of target genes, and to have no false-negatives if the cluster’s size is larger than or equal to the number of target genes. These cases can be marked on the plots starting from the bottom left corner for smallest clusters, and then as clusters include more genes, their marks go up along the vertical axis to the top left corner, and then slide along the black curve towards the bottom right corner. Almost all of the cases of the cluster C1 generated by our method occur on the aforementioned path and large portions of the cases of the cluster C2 also occur on that path as well (Fig. 4). Moreover, in many cases, our method has been successful in finding the theoretically ideal cluster; this has happened at almost all of the genome sizes for C1 and for the first two genome sizes for C2. The *K* and δ or (δ^+^, δ^−^) values at which the best clusters were found for each of the considered genome sizes are shown in Table 2.

**Table 2:** UNCLES parameters for the clusters selected by the M-N scatter plots for types A and B at each of the considered GS values

Cluster	GS	K	δ	Cluster	GS	K	δ+	δ-
C1	1,200	4	0.8	C2	1,200	4	0.7	0.8
	2,000	4	0.7		2,000	8	0.6	0.7
	3,000	12	0.4		3,000	4	0.7	0.8
	5,000	4	0.7		5,000	20	0.3	0.6
	7,000	4	0.8		7,000	8	0.5	0.8

Most of the clusters generated by our method in both cases are irrelevant to the target clusters, i.e. they include no true-positives, and they are shown as dense points at the bottom right corners. Having high densities on the vertical axis, the black continuous carve, and the bottom right corner, with low densities elsewhere, indicates that the results clearly separate the relevant cluster with its different tightness levels from the rest of the irrelevant clusters.

There is general agreement between the ground-truth-independent approach (M-N plots) and the ground-truth-dependent approach (F-P plots). Slight perturbations in the ground-truth-independent approach (M-N plots) have been seen to lead to such slight perturbations in the ground-truth-dependent approach (F-P plots). This demonstrates the robustness of our approach in selecting the best cluster in an independent manner of the known ground-truth, i.e. the M-N plots approach.

To assess the uniqueness of type B further, we have applied UNCLES type A only to the datasets P1, P2, and P3. As expected, and at all of the considered GS values, two distinctly and equally high-quality clusters were identified in this supplementary experiment representing the clusters C1 and C2. Indeed both clusters are consistently co-expressed in those three datasets. However, type B filters out the cluster C1 because it requires an additional specification to be satisfied, that is, for the cluster to be poorly co-expressed in the negative datasets N1, N2, and N3.

**Fig. 5:**
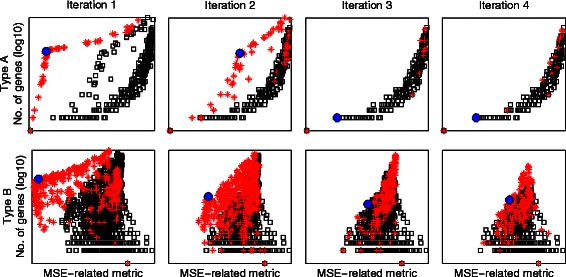
Demonstration of the iterative process of selecting the best four clusters from both types *A* and *B* using *M-N* plots while analysing the synthetic datasets with a *GS* of 1,200. The union of the scattered black squares and red stars in the *M-N* plots of the first column represents all of the clusters generated at all of the *K* values and at all of the δ or (δ+, δ-) values. The big solid blue circle represents the best cluster, i.e. the cluster closest to the top left corner. The red stars represent the clusters which share at least one gene with that best cluster. Moving through the plots from the left to the right, the clusters marked by red stars are removed and the process is repeated iteratively over the remaining clusters. The first four iterations for types A and B are shown in this Figure

For a further demonstration of the iterative process of the M-N plots, we show the M-N plots for the first four iterations for both types A and B while analysing the set of datasets with GS = 1,200 in Fig. 5. The plots in the first iteration, represented by the leftmost column in this Figure, are identical to those shown in Fig. 4. The best cluster is shown as a solid blue circle, the clusters which share at least a single gene with that best cluster are shown as red stars, while the rest of the clusters are shown as black squares. The plots in the second iteration represent those from the first iteration after removing the best cluster and all of those clusters which have some overlap with it, i.e. the solid blue circle and the red stars. The closest cluster to the top left corner in the reduced M-N plot is identified as the second best cluster, and the iterative process is repeated again. More about the stopping criteria of this process are discussed and demonstrated in the analysis of budding yeast data later in this article.

#### Weighting datasets by numbers of samples

In order to assess the effect of differences between datasets in their number of samples / data-points, we have repeated the experiment while weighting the datasets by their numbers of samples. Weighting takes place at the stage of combining the relabelled individual partitions produced by clustering each dataset separately by multiple methods. More precisely, combining those partitions takes the form of weighted averaging instead of ordinary averaging in order to produce the fuzzy consensus partition matrix (CoPaM). In this case, the fuzzy membership value of a given gene in a given cluster is a weighted contribution of all datasets proportional to their relative numbers of samples.

**Fig. 6:**
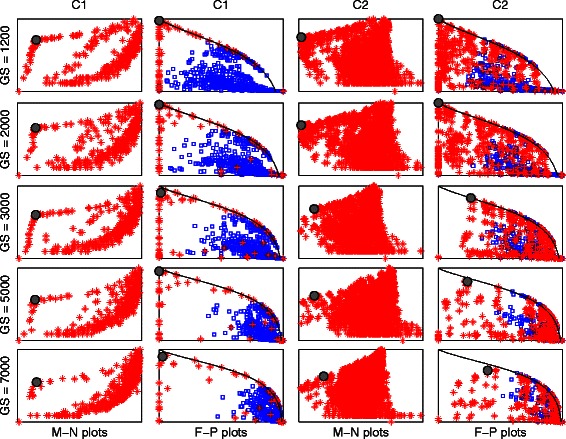
M-N and F-P scatter plots of the synthetic data clusters *C1* and *C2* generated by *UNCLES,* weighted by datasets’ numbers of samples, and by other methods. The selected clusters in the M-N plots are marked by solid grey circles, and their corresponding points in the F-P plots are marked by solid grey circles as well. The red stars in any of the *M-N* or *F-P* plots represent the clusters produced by the UNCLES method while the blue squares in the F-P plots represent the clusters produced by the other methods

Fig. 6 shows the M-N scatter plots and the F-P scatter plots resulted from this experiment in a similar format to Fig. 4. It can be seen while comparing the two Figures that the results are very similar. It is worth mentioning that the number of samples in the six datasets P1, P2, P3, N1, N2, and N3 is 18, 18, 6, 16, 12, and 12, respectively. Taken together, it has been demonstrated that the results of the UNCLES analysis do not change significantly by weighting the datasets by the number of samples in this given range.

#### Robustness to gene expression perturbations

Measured gene expression values are composed of the actual gene expression in addition to various undesirable components such as noise due to the inaccuracy of the biological setup of the experiments or the technical tolerance of the technologies adopted (e.g. microarrays). Thus, if the same biological experiment was performed multiple times to measure the expression of the same genes, it is expected that the measured values will vary around a mean value representing the actual expression.

We have undertaken an experiment to test the robustness of our method’s results under such variations. This has been done by adding a zero-mean Gaussian noise to the expression values of all of the genes in all of the six datasets and then applying UNCLES followed by the M-N plots technique to them. The standard deviation of the added Gaussian noise has been estimated for each sample / time-point in each of the datasets independently and based on the data itself. Let the standard deviation at the sample / time-point *i* of the dataset *d* be *σ*_*di*_, where *d* ∈ {*P*1, *P*2, *P*3, *N*1, *N*2, *N*3} and *i* ∊ [1…*N*_*d*_] such that *N*_*d*_ is the number of samples / time-points in the dataset *d*. The value of *σ*_*di*_ is estimated by the following equation:

4 $$ {\sigma}_{di}=\left\{\begin{array}{c}\hfill \left(\sqrt{\frac{{\displaystyle {\sum}_{g\in C1}}{\left({x}_{dig}-{\overline{x}}_{diC1}\right)}^2}{75 - 1}}+\sqrt{\frac{{\displaystyle {\sum}_{g\in C2}}{\left({x}_{dig}-{\overline{x}}_{diC2}\right)}^2}{85-1}}\right)/2,\ d\in \left\{P1,\ P2,\ P3\right\}\ \hfill \\ {}\hfill \sqrt{\frac{{\displaystyle {\sum}_{g\in C1}}{\left({x}_{dig}-{\overline{x}}_{diC1}\right)}^2}{75-1}},\ d\in \left\{N1,\ N2,\ N3\right\}\hfill \end{array}\right., $$

where *x*_*dig*_ is the genetic expression of the gene *g* at the sample / time-point *i* in the dataset *d* and ${\overline{x}}_{diC1}$ and ${\overline{x}}_{diC2}$ are the mean expression values of the genes in the clusters C1 and C2 respectively at the sample/time-point *i* in the dataset *d*.

The justification of this modelling of the standard deviation of the added noise is that the genes in the cluster C1 are assumed to be co-expressed as per the studies which produced the six datasets, and the genes in the cluster C2 are assumed to be co-expressed as per the studies which produced the positive datasets P1, P2, and P3 (see Methods for details). However, the expression profiles of the genes within those clusters are not identical and do vary from each other due to the aforementioned factors. Therefore, we consider that the standard deviation observed in the genes within those clusters at a given sample/time-point is representative of the possible variation of any given gene at that sample/time-point if measured multiple times under similar conditions. It is worth noting that the datasets already have noise as they have been assembled from real datasets and the added noise is an extra layer of noise to test the method’s robustness further.

**Fig. 7:**
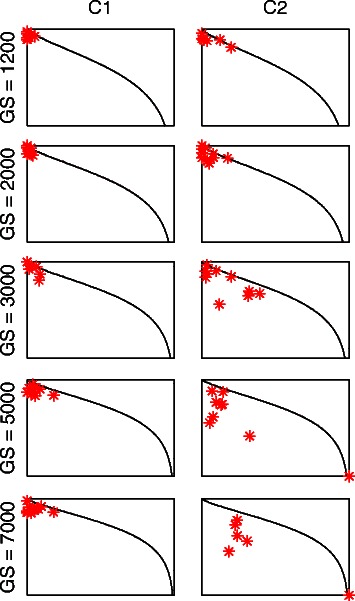
*F-P* scatter plots of the best *C1* and *C2* clusters selected by the M-N plots after applying UNCLES types *A* and *B* to the synthetic datasets with added noise. Each sub-plot is an *F-P* scatter plot with ten points representing the best *C1* or *C2* cluster identified by each of the ten repetitions of the experiment of adding noise to the datasets, clustering by the UNCLES method, and cluster selection by the M-N scatter plots. This experiment has been performed for each of the five different adopted genome-sizes from 1,200 to 7,000. The scale of each F-P plot, which has been omitted for clarity, is from zero to unity for both dimensions

We have repeated this experiment of adding Gaussian noise to the datasets followed by applying the UNCLES method and the M-N scatter plots technique ten times for each of the considered genome-sizes from 1,200 to 7,000. The first cluster selected by the M-N scatter plots in each of the ten repetitions given an UNCLES type (A (cluster C1) or B (cluster C2)) at a given genome-size (GS) is plotted as a point on the relevant F-P scatter plot in Fig. 7. Each sub-plot in this Fig. is related to a given UNLES type and a GS value, while the ten points plotted in any of those sub-plots represent the top clusters in each of the ten repetitions of the experiment.

It can be seen in this Figure that, despite the added noise, the results of type A (C1) at all GS values and the results of type B (C2) at GS values up to 3000 are extremely close to the ideal result represented by the top-left corners of the F-P plots. Nonetheless, the results of type B (C2) at high GS still show very good proximity from the top-left corners in most of the repetitions, relative to those in Fig. 4. This experiment strongly demonstrates the robustness of the UNCLES method coupled with the M-N scatter plots under extra levels of noise.

#### Comparison with biclustering methods

Biclustering methods aim at finding genes that are co-expressed, not necessarily in all of the provided data samples, but at least in some of them. A bicluster is a cluster defined by a subset of genes and a subset of data samples (data matrix columns). Here, we compare our UNCLES analysis of the synthetic datasets with eight different biclustering methods.

**Fig. 8:**
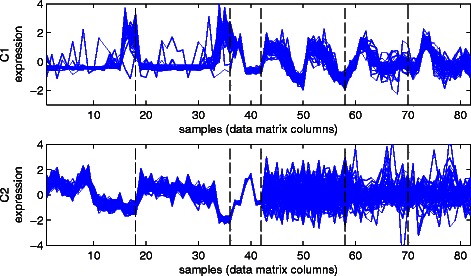
Synthetic data ground truth clusters *C1* and *C2* combined expression profiles from all of the six datasets. The vertical dashed lines show the boundaries between the samples belonging to each of the six datasets in their respective order of *P1, P2, P3, N1, N2,* and *N3. C1* shows consistent co-expression over all of the combined 82 samples (data matrix columns), while *C2* shows consistent co-expression only over the first 42 samples

Biclustering methods can be applied only to a single dataset. Therefore, and given any genome size (*GS*), we have concatenated the six synthetic datasets horizontally to produce a single data matrix with *GS* rows and 82 columns, where this number of the columns is the total number of columns (samples) in all of the six datasets. The profiles of the two ground-truth clusters C1 and C2 in the combined dataset are shown in Fig. 8. The first 42 columns belong to the three positive datasets P1, P2, and P3, while the last 40 columns belong to the three negative datasets N1, N2, and N3, and it can be clearly seen in this Figure that C1 genes are consistently co-expressed in all of the 82 columns (samples) while C2 genes are distinctly co-expressed in the first 42 ones.

Eight different biclustering methods were applied to the combined datasets, namely Cheng and Church (CC) [15], Plaid [16], Bimax [17], Spectral [33], FLOC [34], XMOTIFS [35], large average sub-matrices (LAS) [36], bipartite spectral graph partitioning (BSGP) [37]. At all genome sizes, Spectral and XMOTIFS produced no clusters, while CC produced a single trivial cluster that encompasses the entire genome and all of the data samples. Comparison between the UNCLES method and the five biclustering methods that neither produced no clusters nor included the entire dataset in a single cluster is shown in Table 3.

**Table 3:** Comparison between UNCLES and eight biclustering methods

Cluster and *s*	UNCLES^ab^	Plaid^a^	Bimax^a^	FLOC^a^	LAS^a^	BGSP^a^
C1 1200	**0.00**	0.10	1.00	1.35	0.13	0.61
	**82/82**	20/82	4/82	6/82	21/82	1/82
C1 2000	**0.00**	0.64	1.06	1.38	0.16	0.75
	**82/82**	22/82	4/82	6/82	21/82	2/82
C1 3000	**0.00**	0.95	1.12	1.39	0.29	0.90
	**82/82**	37/82	4/82	6/82	18/82	0/82
C1 5000	**0.04**	1.28	1.21	1.40	0.45	0.06
	**82/82**	5/82	3/82	6/82	18/82	0/82
C1 7000	**0.02**	0.97	0.95	1.40	0.59	0.09
	**82/82**	30/82	4/82	6/82	19/82	0/82
C2 1200	**0.00**	0.76	1.21	1.36	0.31	0.96
	**42/42**	5/42	3/42	2/42	15/42	0/42
C2 2000	**0.00**	0.92	1.26	1.37	0.28	0.91
	**42/42**	16/42	3/42	3/42	15/42	0/42
C2 3000	0.33	0.99	1.29	1.38	**0.32**	1.00
	**42/42**	5/42	3/42	5/42	15/42	0/42
C2 5000	**0.40**	1.07	1.32	1.40	0.71	1.14
	**42/42**	5/42	3/42	2/42	13/42	0/42
C2 7000	**0.43**	1.18	1.30	1.40	0.70	1.17
	**42/42**	5/42	3/42	4/42	13/42	0/42

^a^Each cell in those columns includes two values – the first is the distance from the top-left corner of the ground-truth-based F-P plots for the best cluster found by each method; the ideal is zero and the maximum is $\sqrt{2}\cong 1.41$; the second value is the number of data samples (data matrix columns) which the algorithms correctly found for the corresponding clusters out of the total number of correct samples (82 for type A and 42 for type B)

^b^The number of data matrix columns (samples) are prefixed for UNCLES while being variable for biclustering methods

Table 3 shows two metrics for each method’s results considering the clusters C1 and C2 based on each of the five different considered genome sizes (*GS*). The first metric is the shortest distance from the top left corner of the F-P scatter plot; this ranges from 0.0 for the ideal cluster to $\sqrt{2}\cong 1.41$ for the worst possible cluster. The second metric is the number of correctly identified data matrix columns (data samples) out of the total number of correct data matrix columns; for type A, all of the 82 samples (combined from the six datasets) represent the correct samples, while for type B, the 42 samples originally belonging to the positive datasets P1, P2, and P3, are the correct ones.

At all genome sizes, and for both types, type A (cluster C1) and type B (cluster C2), the UNCLES results showed the best performance (minimising the distance and maximising correctly identified data matrix columns / samples). The only exception is for C2 at the genome size (*GS*) of 3,000 genes, where the LAS method scores a subtly smaller distance than UNCLES. However, even at that latest case, UNCLES’ F-P distance is 0.33 compared to 0.32 for LAS, which indicates an insignificant difference between the two distances. Moreover, LAS and all of the other biclustering methods have identified only few data matrix columns out of the total number of correct columns.

Although all of the biclustering methods lag behind UNCLES, it can be seen that Plaid, LAS, and BSGP, perform relatively better than FLOC and Bimax. In general, LAS shows more consistent quality across varying genome sizes (*GS*) compared to Plaid and BSGP.

### Budding yeast data analysis

#### Data and experimental setup

We have analysed two subsets of budding yeast datasets (Table 4). The positive subset (S^+^) consists of eight yeast cell-cycle datasets [38–41]. Each of these eight datasets represents the genetic expression of the budding yeast (*Saccharomyces cerevisiae*) genome over two cell-cycles. The negative subset (S^−^) consists of six non-cell-cycle budding yeast datasets [42–44]. We found 4422 genes which are included in each of 14 datasets and meet the allowed missing values criterion (Table 4); these were the genes to which we have applied our analysis.

**Table 4:** Budding yeast microarray datasets

Name	Genes	Time pts.	Total time (min)	Missing values allowed	Reference
S+					
Cdc28	6223	17	160	1 / 17	[39]
Alpha	6178	18	119	1 / 18	[38]
Alpha-30	6266	25	120	1 / 25	[40]
Alpha-38	6266	25	120	1 / 25	[40]
Orl-wt1	5667	15	224	0/15	[41]
Orl-wt2	5667	15	224	0/15	[41]
Orl-mt1	5667	15	224	0/15	[41]
Orl-mt2	5667	15	224	0/15	[41]
S-					
Sporulation	6118	7	690^a^	0/7	[42]
C-impulse	5667	15	420^a^	0 / 15	[44]
N-impulse	5667	15	420^a^	0 / 15	[44]
MMS-wt	6127	7	120^a^	1 / 7	[43]
Gamma-wt	6127	8	120^a^	1 / 8	[43]
Mock-wt	6127	4	90^a^	0 / 4	[43]

^a^The time-points for these datasets were not sampled uniformly over the total time interval

Most of the datasets were normalised by the groups who generated them in a manner which suits the nature of the microarray chips used to produce them. However, we have also ensured that the genes of all of the datasets have a zero mean, and as recommended by the review by Roberts [31], we have further normalised the one-channel datasets by quantile normalisation and let them have a unity standard deviation.

While considering k-means with KA initialisation [7], SOMs [9], and HC with Ward’s linkage [8] as starting methods, we have applied UNCLES with both types A and B of external specifications to these datasets. These two types can be restated as finding the subsets of genes that are generally consistently co-expressed in budding yeast under various conditions and different biological contexts for type A, and finding the subsets of genes that are specifically consistently co-expressed in yeast cell-cycles while losing such consistency under other biological conditions for type B. These other conditions include sporulation, carbon and nitrogen nutrient perturbation, and stress conditions (Table 4). The adopted numbers of clusters (K) have been 4, 8, 12, 16, 20, and 25 while the values of δ (type A), δ^+^, and δ^−^ (type B), range from zero to unity with steps of 0.1. Therefore, there are 935 resulting clusters from type A and 10,285 clusters from type B.

#### Clusters evaluation and selection

**Fig. 9:**
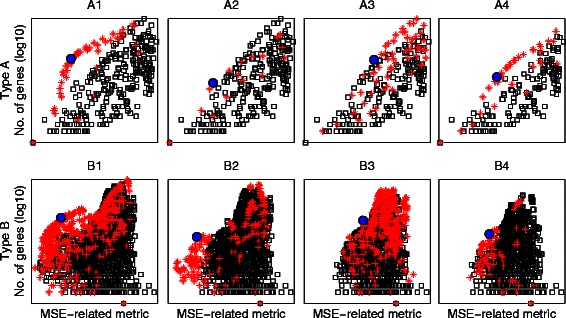
Demonstration of the iterative process of selecting the best four yeast clusters from both types *A* and *B* using *M-N* plots. The union of the scattered black squares and red stars in the *M-N* plots of the first column represents all of the clusters generated at all of the K values and at all of the δ or (δ+, δ-) values. The big solid blue circle represents the best cluster, i.e. the cluster closest to the top left corner. The red stars represent the clusters which share at least one gene with that best cluster. Moving through the plots from the left to the right, the clusters marked by red stars are removed and the process is repeated iteratively over the remaining clusters. The first four iterations for types A and B are shown in this Figure

The M-N scatter plot for the 935 clusters of type A is shown in the sub-plot (A1) in Fig. 9. The closest cluster to the top-left corner is selected as the best cluster and marked by a solid blue circle. All of the clusters which share at least a single gene with A1 are considered as other versions of it, are marked by red stars, and are then excluded from the complete set of clusters. The next M-N plot of type A shows the same clusters of the first M-N plot after the exclusion of the best cluster and the other versions of it, i.e. after excluding the solid blue circle and the red stars. The best cluster for that second iteration is selected by the same approach, named as A2, and the process is repeated iteratively. Fig. 9 shows the M-N plots for the first four iterations while selecting the best clusters of both types A and B. Indeed, type B clusters are labelled as B1, B2, etc.

**Fig. 10:**
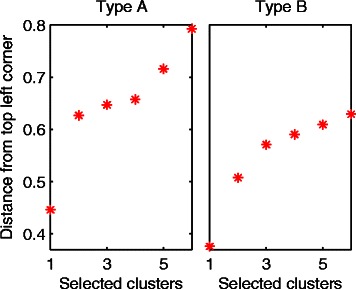
Distances from the top left corners of the M-N plots for the yeast clusters selected at the first six iterations for types *A* and *B*

**Fig. 11:**
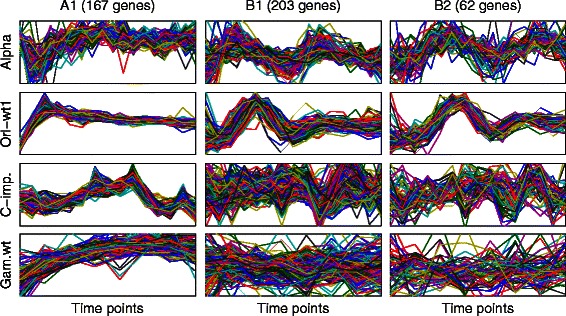
The normalised genetic expression profiles of the genes included in the selected yeast clusters from both UNCLES types *A* and *B* in two S+ and two S- representative datasets

Fig. 10 shows the distances of the selected clusters at the first six iterations for both types A and B from the top-left corners of the corresponding M-N scatter plots (the M-N plots for the first four iterations are shown in Fig. 9). It can be seen for type A that there is a large gap between the first cluster (distance = 0.45) and the second cluster (distance = 0.63). Therefore, we have selected the cluster A1 as the only significant cluster for type A. Although the same scenario can be seen in type B (distance of B1 = 0.38, and distance of B2 = 0.51), there is another gap between the second and the third clusters (distance for B3 = 0.57). We have selected both clusters B1 and B2 as the significant clusters of type B. It can be clearly seen in Fig. 11 that A1 is consistently co-expressed in all of the four datasets while B1 and B2 are exclusively consistently co-expressed in the two representative S^+^ datasets while being poorly co-expressed in the two representative S^−^ datasets. Thus, the results match the original different external specifications set for both types A and B.

Some researchers might choose to select more clusters based on Fig. 10 than those we have chosen. We consider this selection to be a study-specific issue on which the decision is made based on the tolerance of cluster quality that best serves the given requirements. For example, one may choose to select the first four clusters in the case of type A because there is a gap in distances between the fourth and the fifth clusters as seen in Fig. 10. However, if a researcher terminates selection of clusters after the third cluster, it can be argued that the fourth cluster is not significantly different from the third for it to be excluded while including the third.

#### Biological information-based validation

We have used the GO Term Finder tool of the Saccharomyces Genome Database to find the enriched biological processes in the clusters A1, B1, and B2 (Additional file 6). The most enriched processes in A1 are ribosome biogenesis (120/167 genes, *p-value* 4.6 × 10^−114^) and many RNA processing processes like ncRNA processing (99/167 genes, *p-value* 3.8 × 10^−80^), rRNA processing (87/167 genes, *p-value* 1.6 × 10^−75^), and RNA processing (99/167 genes, *p-value* 1.4 × 10^−63^). The most enriched processes in B1 are DNA metabolic process (65/203 genes, *p-value* 3.3 × 10^−26^), cell cycle (74/203 genes, *p-value*7.8 × 10^−25^), and many other processes related to DNA metabolism such as DNA replication (34/203 genes, 1.2 × 10^−19^) and DNA repair (41/203 genes, 6.4 × 10^−19^). The most enriched processes in B2 are chromosome organisation (21/62 genes, 8.4 × 10^−9^), microtubule-based process (12/62 genes, 1.1 × 10^−7^), mitotic cell cycle (17/62 genes, 5.8 × 10^−7^), and chromosome segregation (12/62 genes, 1.2 × 10^−6^).

Note that genes which participate in ribosome biogenesis and RNA processing processes have been previously reported to be generally co-expressed in various types of conditions, and that they are generally up-regulated under growth conditions but down-regulated under stress conditions [6, 11]. This observation validates the identification of this subset of genes (A1 in our results) as the most consistently co-expressed subset of genes in budding yeast over 14 different datasets. It can be clearly seen that the clusters B1 and B2 are both enriched with cell cycle-related processes, and that their profiles in the cell cycle datasets are cyclic (Fig. 11). These facts resonate well with the original question which has been addressed by type B of our novel method. It can also be seen in Fig. 11 that there is a phase shift between the cyclic profiles of B1 and B2. By referring to the studies which generated these datasets (Table 4), it can be seen that the cluster B1 peaks at the transition between the stages of gap 1 and synthesis (G1/S transition) while B2 peaks at late synthesis stage (S). The enriched biological processes in both clusters are consistent with this observation [38, 40].

#### Genes with unknown biological processes in A1 may be involved in rRNA processing and ribosome biogenesis

There are seven, out of the 167, genes in A1 that have unknown biological processes (GO Slim analysis in Additional file 6). The seven genes are YBL028C, BMT2, YCR016W, RRT14, CMS1, TMA16, and YDR514C. Despite not being assigned to a known biological process, many observations in the literature, as detailed below, resonate well with their inclusion in this cluster enriched with rRNA processing and ribosome biogenesis genes. In terms of localisation, all seven genes’ products are localised in the nucleus (p.v. 1.7 × 10^−3^) while the first four are also localised in the nucleolus (p.v. 6.4 × 10^−3^), where ribosome biogenesis actually occurs. YBL028C and TMA16 were found to co-localise with the ribosome [45]. YBL028C, YCR016W, and RRT14 are amongst the hundreds of genes predicted by Wade and colleagues to be involved in ribosome biogenesis, but never confirmed [6]. BMT2 has been found to methylate adenine (m1A) of the large subunit (LSU) rRNA [46], and CMS1 is a putative subunit of the 90S preribosome processome complex [47].

To investigate co-regulation, we have used the MEME tool to analyse the 300 DNA upstream base-pairs of the genes included in this cluster [48]. The top two discovered motifs were found in the upstream sequences of 149 and 133 out of 167 genes in A1 respectively with the respective E-values of 1.2 × 10^−334^ and 5.9 × 10^−112^. By using the TOMTOM tool, the first of motif was found to match the binding sites of the transcription factors DOT6 (p.v. 6.9 × 10^−6^) and TOD6 (p.v. 2.4 × 10^−4^). The second motif matches the ribosomal RNA processing element (RRPE), which is the binding site of STB3 (p.v. 3.1 × 10^−6^). Those transcription factors are well known regulators of the rRNA processing and ribosome biogenesis regulon [6, 49]. The first of those two motifs was found in the upstream sequences of six out of the seven genes with unknown processes, namely all but YCR016W, while the second one was found in four of them, namely YBL028C, BMT2, YCR016W, and TMA16.

In conclusion, those observations indicate that six out of the seven genes with unknown biological processes in A1, after excluding YCR016W, may be involved in ribosome biogenesis and/or rRNA processing, and that they are co-regulated with them.

#### Genes with unknown biological processes in B1 may be involved in the G1/S cell-cycle phase

The GO Slim analysis conducted in this study has revealed that 24 out of the 203 genes included in the cluster B1 have not been assigned to any known biological process (Additional file 6). As this cluster shows a cyclic expression which peaks at the G1/S phase transition of the cell-cycle (Fig. 11), we have compared its contents with the cluster C1 identified and thoroughly investigated in our recent study [18]. Interestingly, 17 out of 19 genes included in C1 at δ = 1.0 are also included in B1 (Table 5). Those include the gene CMR1 (YDL156W) which was the main subject of that recent study. Moreover, B1 includes more than half of the genes included in C1 at all tightness levels. More importantly, it virtually includes all of the genes hypothesised in that study to be co-working with CMR1 such as the three subunits of the replication factor A (RFA1, RFA2, and RFA3) and most of the subunits of the DNA polymerases [18].

**Table 5:** Comparison between the B1 cluster in this study and the C1 cluster in our previous study [18] at varying δ values

C1 δ value	Total in C1	Also in B1	Not in B1
0.0	216	113	103
0.95	148	90	58
0.99	117	81	36
1.0	19	17	2

B1 includes 203 genes, 90 of which are not included in C1 even at δ = 0

More interesting findings have been observed when we investigated the GO term enrichment in the 90 genes included in B1 but not in C1 at any of its levels of tightness as well as the 103 genes included in C1 even at δ = 0.0 but not in B1. We will refer to those two subsets of genes by using the set difference notation (B1 – C1) and (C1 – B1), respectively. The (B1 – C1) subset is enriched with the terms “cell-cycle process” (32/90 genes; p.v. 2.3 × 10^−10^), “DNA metabolic process” (25/90 genes; p.v. 1.3 × 10^−7^), and other related processes, while 12 out its 90 genes have unknown biological processes. On the other hand, there are 41 genes, out of the 103, in (C1 – B1), that have unknown biological processes, and the most enriched known biological processes are “telomere maintenance via recombination” (7/103 genes; p.v. 1.9 × 10^−6^), “DNA recombination” (13/103 genes; p.v. 2.1 × 10^−4^), and “DNA metabolic process” (20/103 genes; p.v. 3.6 × 10^−3^). Thus, B1 is more focused than C1 on the processes of interest in both studies, i.e. cell-cycle and DNA metabolism processes.

We have also used the MEME tool to identify the most enriched motifs in the upstream sequences of the genes in B1. The top two motifs were found in the upstream sequences of 179 and 117 genes with the E-values of 2.2 × 10^−109^ and 5.0 × 10^−64^, respectively. By using the TOMTOM tool, the first motif was found to match binding sites of the transcription factors AZF1 (p.v. 1.1 × 10^−5^) and SFL1 (p.v. 3.9 × 10^−4^). Interestingly, the second motif was found to match the binding sites of the transcription factors SWI4 (p.v. 1.8 × 10^−5^) and MBP1 (p.v. 4.4 × 10^−5^), and the binding site of the transcription factor complex MBP1/SWI6 (p.v. 9.0 × 10^−5^). Those later transcription factors are well known regulators of the cyclic genes peaking at the G1/S transition [50, 51, 18], which is consistent with our findings.

Taken together, these findings and comparisons clearly show that the new approach reconfirms the hypotheses presented in our previous study regarding the gene CMR1 (YDL156W). This study redefines the subset of genes peaking at G1/S transition which may be involved in cell-cycle and DNA metabolism processes. Therefore, we hypothesise that the 24 genes included in this cluster with unknown processes may be involved in the cell-cycle G1/S phase progression through DNA metabolism, and that they are expected to be co-regulated with the other known genes in this cluster.

## Discussion

We have proposed a new method, UNCLES, which unifies the results of clustering analysis of multiple datasets based on different types of external specifications. Although the main context of this study considers transcriptomic datasets (e.g. microarray datasets), any other set of datasets over which analogous questions can be asked are subject to our method. We have defined two types of external specifications; type A mines for the subsets of genes consistently co-expressed in all of the included datasets, and type B mines for the subsets of genes specifically consistently co-expressed in one subset of datasets (S^+^) while being poorly consistently co-expressed in another subset of datasets (S^−^). We have also proposed a novel technique to solve the problem of selecting the best cluster(s) out of all of the generated results by both types of UNCLES at all of the different tightness values. This novel technique, which is based on the proposed M-N scatter plots, therefore solves the problems of setting the best number of clusters (K) as well as the tuning parameters δ and (δ^+^, δ^−^). Finally, our analysis of the real yeast datasets has resulted in drawing *in silico*-based hypotheses which identify potential biological processes of a subset of genes with previously unknown processes.

### UNCLES types A and B objectives

Our results have demonstrated the unique ability of UNCLES to address the problem of identifying co-expressed or not co-expressed elements across multiple datasets. This has been done by two comprehensive sets of experiments analysing synthetic datasets and real yeast datasets, respectively. Type A is implemented by configuring the recently proposed Bi-CoPaM method [10], while type B is implemented by a sophisticated combination of a pair of type A results.

Previously, Piro [3] and Choi [13], and their respective colleagues, used network-based approaches to identify the genes which have differential co-expression between different types of datasets. Both studies’ approaches have parts which cannot be applied in the absence of prior knowledge on genes’ functions and roles. UNCLES can therefore be clearly contrasted from those methods in that it is completely unsupervised and only depends on the expression values included in the datasets.

Nilsson [2], Wade [6], and their collaborators started with specific subsets of well-known core genes as templates then mined multiple datasets for genes that consistently match the starting template [6, 2]. The two studies’ diverged in terms of the observed consistency of co-expression of the core genes over the datasets; Wade and colleagues observed consistent co-expression of their core ribosome and rRNA biosynthesis (RRB) genes under various conditions [6], while Nilsson and colleagues observed the specific consistency of co-expression of their core haem biosynthesis genes in blood-related datasets while being poorly co-expressed elsewhere [2]. These studies’ statements, observations, and conclusions prove the importance of addressing the two different questions addressed by the UNCLES method. Furthermore, although those studies have raised those questions, they did not provide a solution to them when they are asked in absence of a well-known template of core genes, and not even when the objective is to find more than one single cluster other than the one which matches such starting template. On the other hand, our results have demonstrated the ability of our method to address those two questions in an unsupervised way.

Other traditional unsupervised methods of co-expression mining, such as the clustering methods k-means [7], self-organising maps [9], and hierarchical clustering [8], and even other consensus clustering methods such as relabelling and voting [32] are statistically and functionally significantly inferior than the UNCLES methods for defining co-expressed subsets of genes across multiple datasets (Fig. 4 and Table 1). These other tested methods were designed to partition all of the genes provided to them into a number of clusters. They do not allow genes to be excluded from all of the clusters and therefore they do not have the ability of our method to start from an entire genome and end at focused subsets of genes.

Contrary to traditional unsupervised clustering methods, biclustering mines a data matrix of rows corresponding to genes and columns corresponding to samples in order to identify biclusters, where each bicluster is identified as a subset of rows (genes) that are well co-expressed in a subset of columns (samples). The identified clusters can overlap in terms of their gene-content as well as sample-content. One of the limiting factors of biclustering methods when compared to UNCLES is that they are only applicable when a single dataset is considered. Therefore, if multiple datasets are considered, they need to be concatenated in order to obtain a single dataset, which requires homogeneity and standardisation. Another major difference between biclustering methods and UNCLES is that UNCLES aims at identifying the genes that are consistently co-expressed in some given datasets or distinctly in a pre-specified subset of them, while biclustering methods aim at identifying the genes that are co-expressed in a variable subset of the given conditions without abiding to a pre-specified subset. In many research instances, the research question naturally specifies the specific conditions in which consistent co-expression is favourable, such as in our analysis of budding yeast data where consistent co-expression has been favourable under cell-cycle conditions in contrast to other conditions. In such cases, UNCLES would be more relevant to be applied.

Beside such fundamental differences between UNCLES and biclustering, performance comparison has shown that UNCLES outperforms biclustering methods in identifying the subsets of genes which meet each of the two types of external specifications, A and B (Table 3). This outperformance applies to both identifying the correct subset of genes as well as identifying the correct subset of data matrix columns (data samples).

### Cluster evaluation using M-N scatter plots

The problems of cluster validation, K value setting, and δ value setting (the parameter for DTB binarisation) were stated in our recent proposal of the Bi-CoPaM method as future work [10]. These problems, as well as the problem of setting the parameter pair (δ^+^, δ^−^) for UNCLES type B, have now been solved by the proposed M-N scatter plots technique for cluster evaluation and selection.

We validated the M-N scatter plots technique by using ground-truth-dependent cluster evaluation metrics. As can be seen in our results (e.g. Fig. 4), the clusters that are deemed to be best based on our proposed M-N scatter plots also score the highest, or very close to the highest, scores in the ground-truth-dependent metrics. Furthermore, the M-N scatter plots technique has been further validated when it was applied to real yeast datasets and provided specific clusters with high biological relevance.

This technique has addressed the problem of the dependency of the MSE metric on the number of genes within the cluster by restating the objective to be obtaining the largest clusters which are still tight, and this particular approach of dealing with clusters has made it very suitable to evaluate the clusters of the UNCLES method which have tunable levels of size and tightness. By applying UNCLES with either type A or B many times with various K values, and then putting all of the resulting clusters in one pool for evaluation and selection by the M-N scatter plot approach, the problem of determining the best K value has been solved. Finally, the M-N scatter plots technique is robust across datasets with different genome sizes, numbers and sizes of the selected clusters and biological contexts. It has been observed that slight variations to the distribution of the clusters on an M-N plot would lead to similar slight variations in the final selected cluster.

### Synthesising datasets based on real data measurements

Our proposed approach for generating synthetic datasets is based on using real data measurements in a controlled manner. The genetic expression profiles in those datasets represent real data expression profiles including all of real data implications, but with artificial gene labels. This approach accommodates both realistic modelling of real data and ground-truth knowledge of synthetic data. This overcomes the problems which normally appear in the mathematical models described previously, that try to mimic real genetic expression and its accompanying noise with least approximation errors [27, 28, 30, 29].

### Summary and conclusions

Our results have demonstrated the unique ability of our proposed method, UNCLES, in answering two research questions with both of its types A and B in an unsupervised and robust manner. We have also proposed and validated a novel M-N scatter plots technique for cluster evaluation. This technique was successful in selecting the best clusters while varying the number of clusters (K value) as well as the δ and (δ^+^, δ^−^) values. Therefore, by integrating this technique with the UNCLES method, the method becomes automated and can proceed from the input set of datasets and individual clustering methods to the final few focused clusters without the need to set any critical parameter. Additionally, we have proposed an approach for expression data synthesis, where although the ground-truth is controlled and known, the actual data measurements are borrowed from real datasets reflecting real rather than artificially modelled values. Those sets of synthetic datasets, which are available in Additional files 1, 2, 3, 4, and 5, have been utilised to validate the UNCLES method and the M-N plots technique while being compared to other conventional clustering, consensus clustering, and biclustering methods. Lastly, we have drawn biological hypotheses, based on *in silico* UNCLES analysis, which relate some budding yeast genes with some biological processes in which they are potentially involved. These hypotheses represent significant pilots for future focused studies. UNCLES has the potential to be expanded by producing more types of external specifications for the unification of clustering results to meet other research requirements. It is also now ready to be adopted by biologists and other scientists to analyse diverse types of datasets.

## Additional files

Additional file 1:
**Synthetic data with the genome-size (GS) of 1,200.**

Additional file 2:
**Synthetic data with the genome-size (GS) of 2,000.**

Additional file 3:
**Synthetic data with the genome-size (GS) of 3,000.**

Additional file 4:
**Synthetic data with the genome-size (GS) of 5,000.**

Additional file 5:
**Synthetic data with the genome-size (GS) of 7,000.**

Additional file 6:
**Results of budding yeast data analysis.**
